# Comparison of short‐term outcomes and perioperative costs in laparoscopic versus robotic surgery for rectal cancers: A real‐world cohort study using Japanese nationwide inpatient database

**DOI:** 10.1002/ags3.12884

**Published:** 2024-11-15

**Authors:** Hiroki Hamamoto, Masato Ota, Takafumi Shima, Toru Kuramoto, Kazuya Kitada, Kohei Taniguchi, Mitsuhiro Asakuma, Yasuhiro Oura, Yuri Ito, Sang‐Woong Lee

**Affiliations:** ^1^ Department of General and Gastroenterological Surgery Osaka Medical and Pharmaceutical University Takatsuki Japan; ^2^ Department of General Surgery Chibune Hospital Osaka Japan; ^3^ Department of Medical Statistics Research and Development Center Osaka Medical and Pharmaceutical University Takatsuki Japan

**Keywords:** a real‐world cohort study, Japanese inpatient database, perioperative costs, propensity score matching, robotic surgery

## Abstract

**Aim:**

Many studies have revealed the benefits of robotic surgery for rectal cancer; however, real‐world data are insufficient. This study aimed to compare the short‐term outcomes and perioperative costs of laparoscopic and robotic surgery for rectal cancer using a real‐world database.

**Methods:**

The data of patients who underwent laparoscopic or robotic surgery for rectal cancer between January 2018 and January 2021 from a nationwide Japanese inpatient database provided by Medical Data Vision Co., Ltd. were analyzed. We performed propensity score matching (PSM) analysis to compare the in‐hospital mortality, morbidity, readmission rate, reoperation rate, length of postoperative stay, and medical costs between the two groups.

**Results:**

We performed PSM analysis on 18 952 eligible patients. After PSM, 1396 patients in the laparoscopic group and 1396 in the robotic group were compared. The robotic group had a lower surgical site infection rate (2.9% vs. 1.5%, *p* = 0.010), lower respiratory failure rate (1.3% vs. 0.6%, *p* = 0.049), and higher operative medical costs (1 291 371 vs. 1 312 462 JPY, *p* = 0.013). The total medical costs of the two groups were comparable (1 862 439 vs. 1 895 822 JPY, *p* = 0.051).

**Conclusions:**

PSM analysis revealed that robotic surgery was associated with better outcomes than laparoscopic surgery in terms of surgical site infection and respiratory failure rates. The operative medical costs of robotic surgery were significantly higher than those of laparoscopic surgery. However, there was no significant difference in the total medical costs between robotic and laparoscopic surgery for rectal cancer.

## Graphical abstract

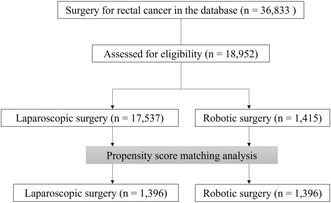

## 1 INTRODUCTION

Colorectal cancer is the third most common cancer worldwide.^1^ Over 700 000 people are newly diagnosed with rectal cancer, and more than 300 000 people die from rectal cancer.^2^

Minimally invasive surgery for rectal cancer has evolved over time and has been widely adopted; however, laparoscopic surgery for rectal cancer (Lap) has difficulties, including the requirement for straight and inflexible devices. Robotic surgery (Ro) for rectal cancer has several advantages over Lap in terms of the use of articulating instruments, enhanced dexterity with tremor filtration, and motion scaling. Weber et al^3^ reported the first case of robot‐assisted colorectal resection in 2002; since then, Ro has been widely used worldwide. The latest randomized controlled trial (RCT) of Ro versus Lap surgery for middle and low rectal cancer (REAL) revealed better short‐term outcomes for Ro than for Lap.^4^ Postoperative complications of Clavien‐Dindo grade II or higher within 30 days after surgery were less frequent in the Ro group than in the Lap group (16.2% vs. 23.1%, *d* = 0.003). After surgery, the patients in the Ro group recovered faster than those in the Lap group, with a shorter time to first flatus, time to first defecation, and postoperative hospital stay. RCTs are performed mainly in specialized centers with extensive surgical experience in eligible patients without complications, and attention should be paid to the adaptation of the results to the general population. However, real‐world data on robotic surgery for rectal cancer are insufficient for evaluating the effectiveness of Ro in real‐world clinical settings.

Several studies^5^, ^6^ using real‐world data have reported that the total cost of Ro is higher than that of Lap. On the other hand, Mizuguchi et al^7^ reported total costs for low‐anterior resection were significantly lower in Ro than Lap (1 955 216 vs. 2 031 511 JPY, *p* < 0.001) using the Diagnosis Procedure Combination (DPC), a large‐scale medical database that records information on hospitalized patients at acute care hospitals in Japan. The study was the first to report that Ro had lower total costs than Lap; however, the authors did not provide details on the operative medical costs.

Thus, this study aimed to clarify the short‐term outcomes and medical costs, including operative costs, of Ro compared with Lap using a nationwide large‐sample dataset.

## 2 METHODS

### 2.1 Study population

We identified patients who underwent Lap or Ro for rectal cancer between January 2018 and January 2021 from the nationwide Japanese inpatient database provided by Medical Data Vision Co., Ltd. We defined the main disease using the International Classification of Diseases (ICD)‐10 codes (C19 and C20) from the database of the main disease names that triggered hospitalization and invested the most medical resources. Patients with missing information (clinical cancer stage, body mass index, or smoking index) were excluded.

### 2.2 DPC system in Japan

The DPC system is a major bundled payment system for medical services applied to inpatients in acute care in Japan.^8^ DPC Data include basic patient information such as age, sex, height, and weight; primary illness at admission; comorbidities at admission; comorbidities that developed during hospitalization; surgeries and procedures performed; all medical resources used; anesthesia time; and discharge outcome. It is a nationwide administrative claims database that covers over 1700 hospitals and 7 million inpatients.^7^ The database is linked to hospitalized patients' insurance information and records total medical costs.

### 2.3 Covariates and outcomes

Body mass index (BMI) was classified into four categories (<18.5, 18.5–24.9, 25.0–29.9, and ≥30 kg/m^2^), and the smoking index was categorized into three categories (0, 1–49, and ≥50 pack‐years). The Charlson comorbidity index was calculated according to Quan's protocol, and each International Classification of Diseases, tenth revision, code for the 17 comorbidities was converted into a score and summed for each patient. The hospital scale was defined as the number of beds, and we created three categories (≤199, 200–499, and ≥500). The clinical stages were graded as 0–I, II, III, or IV. The tumor location was classified as rectosigmoid cancer (C19) or rectal cancer (C20). Intraoperative data included the type of surgical approach (laparoscopic or robotic), creation of a stoma, duration of anesthesia, and blood transfusion. Because data on operative time and intraoperative blood loss were not available from the DPC data, the duration of anesthesia and blood transfusion volume were used as surrogate information.

Postadmission complications were distinguished based on admission comorbidities. The patient outcomes included in‐hospital mortality, morbidity, length of postoperative stay, reoperation during the same admission period, 30‐day readmission, and medical costs. Morbidities included anastomotic leakage (T813), surgical‐site infection [(SSI), T793, T813, T814, T941], peritoneal abscess (K65), bleeding (K661, R58, T810, T811), ileus and bowel obstruction (K560, K562, K565‐567, K913), sepsis (A021, A227, A241, A267, A282, A327, A394, A40, A41, A548, B007, B349, B377), respiratory failure (J12‐18, J690, J691, J958, J959, J96), pulmonary embolism (I26), acute coronary syndrome (I21‐25), stroke (I60‐66), acute renal failure (N17), and urinary tract infection (N10, N30, N390). ICD‐10 codes belonging to multiple classifications were assigned to appropriate complications by reviewing the text. Urinary dysfunction was determined based on whether the patient underwent urethral catheterization, intermittent voiding procedures, or medical therapy (alpha1‐receptor blockers, cholinergic drugs, or cholinesterase inhibitors) on or after the first postoperative day. Medical costs were calculated as total hospitalization and operative medical costs.

Informed consent was not required because of data anonymity. This study was approved by the Institutional Review Board of Osaka Medical and Pharmaceutical University (approval number: 2020‐072‐2).

### 2.4 Statistical analysis

Clinical data and postoperative outcomes were compared between the two groups. Propensity score matching (PSM) was used to match patients who underwent Ro with those who underwent Lap. We used a logistic regression model to calculate the propensity scores. The model was based on the following potential confounding variables: sex, age, BMI, smoking index, Charlson comorbidity index, hospital scale (number of beds), hospital type (designated cancer hospital or not), tumor location, cancer stage, year of operation, and surgical methods. Standardized differences were calculated to compare patient confounders between the Lap and Ro groups. We used nearest‐neighbor matching with a caliper width equal to 0.2 of the standard deviation of the logit of the propensity scores. Categorical variables were compared using the chi‐square test, and for continuous variables the Mann–Whitney *U* test was employed. The significance level was set at *p* < 0.05 for all statistical tests, and all *p* values were 2‐sided. All the analyses were performed using STATA v. 17 (StataCorp, College Station, TX, USA).

## 3 RESULTS

### 3.1 Propensity score matching

**FIGURE 1:**
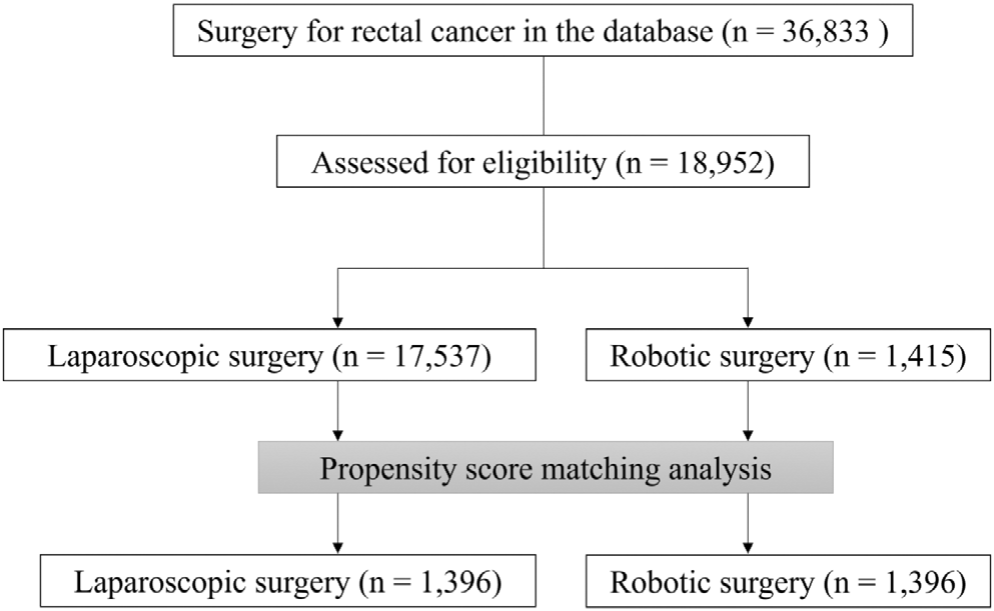
Study flowchart.

A total of 18 952 patients were analyzed in this study and a flow chart is depicted in Figure 1. We identified significant differences in age, smoking index, Charlson comorbidity index, number of beds, designated cancer hospital, tumor location, clinical stage, and PSM was then performed. The baseline characteristics in the two groups were closely balanced by PSM, resulting in 1396 matched pairs (Table 1).

**TABLE 1:** Baseline characteristics.

	Before PSM			After PSM		
	Lap (*n* = 17 537)	Ro (*n* = 1415)	*p* value	Lap (*n* = 1396)	Ro (*n* = 1396)	*p* value
Sex (%)			0.87			0.22
Male	11 253 (64.2)	905 (64.0)		923 (66.1)	892 (63.9)	
Female	6284 (35.8)	510 (36.0)		473 (33.9)	504 (36.1)	
Age (%)			<0.001*			0.70
< 79	14 772 (84.2)	1245 (88.0)		1244 (89.1)	1226 (87.8)	
≥ 80	2765 (15.8)	170 (12.0)		152 (10.9)	170 (12.2)	
BMI (%)			0.27			0.060
<18.5	1842 (10.5)	131 (9.3)		121 (8.7)	130 (9.3)	
18.5–24.9	11 387 (64.9)	911 (64.4)		944 (67.6)	897 (64.3)	
25.0–29.9	3610 (20.6)	309 (21.8)		291 (20.8)	305 (21.8)	
≥ 30	698 (4.0)	64 (4.5)		40 (2.9)	64 (4.6)	
Smoking index (pack years)			<0.001*			0.93
0	8590 (49.0)	624 (44.1)		615 (44.1)	624 (44.7)	
1–49	6416 (36.6)	528 (37.3)		537 (38.5)	528 (37.8)	
≥50	1766 (10.1)	121 (8.6)		127 (9.1)	121 (8.7)	
Unknown	765 (4.4)	142 (10.0)		117 (8.4)	123 (8.8)	
Charlson comorbidity index			<0.001*			0.63
0	6234 (35.5)	699 (49.4)		688 (49.3)	680 (48.7)	
1	4107 (23.4)	289 (20.4)		269 (19.3)	289 (20.7)	
≥2	7196 (41.0)	427 (30.2)		439 (31.4)	427 (30.6)	
Number of beds (%)			<0.001*			
≤199	790 (4.5)	1 (0.1)		1 (0.1)	1 (0.1)	0.56
200–499	9908 (56.5)	276 (19.5)		299 (21.4)	276 (19.8)	
≥500	6839 (39.0)	1138 (80.4)		1096 (78.5)	1119 (80.2)	
Designated cancer hospital			<0.001*			0.18
Yes	13 367 (76.2)	1330 (94.0)	(76.2%)	1327 (95.1)	1311 (93.9)	
No	4170 (23.8)	85 (6.0)		69 (4.9)	85 (6.1)	
Location (%)			<0.001*			0.55
RS	3670 (20.9)	131 (9.3)		122 (8.7)	131 (9.4)	
Ra/Rb	13 867 (79.1)	1284 (90.7)		1274 (91.3)	1265 (90.6)	
Clinical stage (%)			<0.001*			0.86
0–2	9242 (52.7)	841 (59.4)		836 (57.9)	827 (59.3)	
3/4	7217 (41.2)	500 (35.3)		495 (35.4)	495 (35.4)	
Unknown	1078 (6.1)	74 (5.2)		65 (4.7)	74 (5.3)	
Surgical procedure (%)			0.13			0.66
HAR/LAR	15 404 (87.8)	1262 (89.2)		1254 (89.8)	1247 (89.3)	
APR	2133 (12.2)	153 (10.8)		142 (10.2)	149 (10.7)	
Diverting stoma (%)	2196 (12.5)	181 (12.8)	0.77	161 (11.5)	177 (12.7)	0.35

*Note*: Values are expressed as median (range).

Abbreviations: APR, abdominoperineal resection; HAR, high anterior resection; Lap, laparoscopic surgery; LAR, low anterior resection; PSM, propensity score matching; Ro, robotic surgery.

* Significant difference between groups; *p* < 0.05.

### 3.2 Short‐term outcomes and medical costs after PSM

The short‐term outcomes and medical costs before and after PSM are presented in Table 2. Following PSM, we identified significant differences in operative medical costs (Lap vs. Ro: 1 291 371 vs. 1 312 462 JPY, *p* = 0.013), surgical site infection (2.9% vs. 1.5%, *p* = 0.010), and respiratory failure rates (1.3% vs. 0.6%, *p* = 0.049). While comparing Lap versus Ro, the readmission rate (2.4% vs. 2.9%, *p* = 0.35), postoperative length of stay (12 vs. 13 days, *p* = 0.20), and total medical costs (1 862 439 vs. 1 895 822 JPY, *p* = 0.051) did not differ significantly.

**TABLE 2:** Short‐term outcomes and medical cost results.

	Before PSM			After PSM		
	Lap (*n* = 17 537)	Ro (*n* = 1415)	*p* value	Lap (*n* = 1396)	Ro (*n* = 1396)	*p* value
Inhospital mortality within 30 days (%)	53 (0.3)	2 (0.1)	0.28	2 (0.1)	2 (0.1)	1.00
Readmission within 30 days (%)	400 (2.3)	41 (2.9)	0.14	33 (2.4)	41 (2.9)	0.35
Reoperation during the same admission (%) the same admission30 d	532 (3.0)	40 (2.8)	0.66	31 (2.2)	40 (2.9)	0.28
Postoperative length of stay	13 (10–20)	13 (10–18)	0.034*	12 (10–18)	13 (10–18)	0.20
Total medical costs (JPY)	1 895 574 (1 684 842–2 348 302) (4–30)	1 896 767 (1 735 360–2 167 022)	0.66	1 862 439 (1 697 152–2 180 288) (4–30)	1 895 822 (1 733 119–2 169 126)	0.051
Operative medical costs (JPY)	1 281 138 (1 201 151–1 384 083)	1 313 209 (1 235 782–1 397 371)	<0.001*	1 291 371 (1 225 291–1 380 817)	1 312 462 (1 235 476–1 397 032)	0.013*
Total morbidities (%)	4774 (27.2)	177 (12.5)	<0.001*	376 (26.9)	345 (24.7)	0.018*
Leakage	629 (3.6)	34 (2.4)	0.004*	44 (3.2)	34 (2.4)	0.25
Surgical site infection	495 (2.8)	22 (1.6)	0.005*	41 (2.9)	21 (1.5)	0.010*
Bleeding	134 (0.8)	7 (0.5)	0.25	9 (0.6)	7 (0.5)	0.62
Ileus	899 (5.1)	85 (6.0)	0.12	95 (6.8)	84 (6.0)	0.40
Respiratory failure	287 (1.6)	8 (0.6)	<0.001*	18 (1.3)	8 (0.6)	0.049*
Urinary tract infection	408 (2.3)	17 (1.2)	0.006*	25 (1.8)	17 (1.2)	0.21

*Note*: Values are expressed as median (range).

Abbreviations: Lap, laparoscopic surgery; PSM, propensity score matching; Ro, robotic surgery.

* Significant difference between groups; *p* < 0.05.

## 4 DISCUSSION

One significant concern regarding Ro is the overall cost of hospitalization, despite evidence that it yields better short‐term outcomes and is more beneficial to patients compared to Lap. Using a real‐world cohort, our study demonstrated that while the operative medical costs for Ro in rectal cancer were significantly higher than those for Lap, the total medical costs did not show a statistically significant difference. The higher costs associated with Ro are largely due to disposable consumables which limit the number of times it can be used. Despite these higher operative costs, the improved short‐term outcomes associated with Ro have eventually led to similar overall costs compared to Lap. Patel et al^9^ also reported similar findings in their retrospective study conducted at a single center in Canada. They observed that implementing a robotic colorectal surgery program at a Canadian tertiary care center resulted in improved clinical outcomes without a significant increase in the cost of care. Specifically, Ro was associated with higher mean operative medical costs than Lap (mean difference (MD); −$2549, 95% confidence interval (CI); −3374 to −1723$, *p* < 0.0001), whereas the mean total costs of care were not significantly different (MD; −$752, 95% CI; −3603–+2099 $, *p* = 0.17).

In this study, the difference in total medical costs was not statistically significant (*p* = 0.051); however, there was a trend suggesting higher costs for robotic surgery. Furthermore, the actual expenses for Ro are likely higher than those estimated under the DPC system due to equipment purchase costs and maintenance expenses, indicating that robotic surgery places a greater financial burden on hospitals compared to laparoscopic surgery. On the other hand, Morelli et al^10^ reported that increased surgeon experience leads to a significant reduction in costs, indicating that the financial burden associated with Ro may decrease as surgeons gain more familiarity with the technique. Our study relied on data from the early stages of insurance coverage for robotic surgery in Japan. Similar to the findings of Morelli et al,^10^ we expect that costs related to Ro will decline as Japanese surgeons continue to accumulate experience with robotic procedures. Additionally, the emergence of new robotic platforms in the 2020s, such as Hinotori (Medicaroid, Kobe, Japan) and Hugo RAS (Medtronic, Galway, Ireland), is fostering increased price competition, which is expected to reduce medical costs in the future.

In Japan, Ro for rectal cancer was covered by the National Health Insurance in 2018. Every hospital had to pay medical costs for the first 10 cases because national insurance was not available for the first 10 cases, which is a Japanese‐specific insurance system. The first 10 cases were not registered in the Japanese nationwide inpatient database, and the 11th and subsequent cases were registered at each hospital.^7^ Therefore, the first phase of the learning curve was not registered in this study, and many hospitals were presumably in the middle of the learning curve, which might have affected the favorable short‐term results.

The present study showed that Ro was associated with a lower SSI rate than was Lap (1.5% vs. 2.9%, *p* = 0.010). SSIs constitute a financial burden owing to prolonged hospitalization, treatment expenses, and medical staff costs.^11^, ^12^, ^13^, ^14^ This study found no significant difference in the length of hospital stay (Lap vs. Ro: 12 vs. 13 days, *p* = 0.20), but the incidence of SSIs in Lap was approximately twice as high as that in Ro, probably contributing to the increased costs of Lap. According to NCI‐CTC v.2.0, SSIs were defined as superficial, deep, and organ/space, and pelvic abscess and anastomotic leakage were termed organ/space SSIs.^15^ In this study, we defined SSIs using ICD‐10 as follows: T793 (posttraumatic wound infection), T813 (disruption of operation wound), T814 (infection following a procedure; abscess of intra‐abdominal, stitch, subphrenic, and wound), and T941 (sequelae of injury of intrathoracic organs), in accordance with our previously reported cohort studies using DPC.^16^ SSIs also included one part of leakage after surgery; although not significantly different (Lap vs. Ro: 3.2% vs. 2.4%, *p* = 0.25), it tended to be lower in Ro, which may be due to the difference in SSI occurrence. This Japanese nationwide inpatient database clearly showed a lower incidence of SSIs in Ro, which is lower than the data from two well‐known RCTs on Ro [the ROLARR trail^17^: 21/236 (8.9%), the REAL trial^4^:18/536 (3.1%)]; however, the grading of SSIs was unclear in this cohort.

In the present study, Ro was associated with a lower rate of respiratory failure in comparison to Lap (1.3% vs. 0.6%, *p* = 0.049). After PMS, baseline characteristics such as BMI, smoking index, and Charlson comorbidity index were well matched without significant differences, and there were fewer respiratory complications with Ro. Although the difference was not statistically significant, this study period was the introduction phase of robotic surgery; thus, surgeons may have hesitated to perform it on patients with respiratory complications.

To the best of our knowledge, three studies have compared the total costs of robotic and laparoscopic surgeries using real‐world data (summarized in Table 3). Halabi et al^5^ and Chen et al^6^ reported that the total cost of Ro is higher than that of Lap. Mizuguchi et al^7^ reported that total costs for low anterior resection were significantly lower in the Ro group than in the Lap group; however, for high anterior and abdominoperineal resections, the difference was not significant. None of these studies clarified surgical costs, and our study is the first to report surgical costs using real‐world data.

**TABLE 3:** Summary of the study comparing laparoscopic and robotic surgery costs for rectal cancer using real‐world data.

Author	Study duration	Database	Number	After PMS	Total costs	Operative costs
Halabi, et al.	2009–2010	NIS in US	Lap: 115 648	NA	Lap < Ro	NA
			Ro: 2143			
Chen, et al.	2008–2012	NIS in US	Lap: 5578	Lap: 551	Lap < Ro	NA
			Ro: 4744	Ro: 551		
Mizoguchi, et al.	2018–2020	DPC in Japan	LAR		Ro < Lap	NA
			Lap: 16 733	Lap: 1992		
			Ro: 2076	Ro: 1992		
			HAR		Lap = Ro	NA
			Lap: 9130	Lap: 357		
			Ro: 368	Ro: 357		
			APR		Lap = Ro	NA
			Lap: 3807	Lap: 310		
			Ro: 341	Ro: 310		
Our study	2018–2021	DPC in Japan	Lap: 17 537	Lap: 1396	Lap = Ro	Lap < Ro
			Ro: 1415	Ro: 1396		

Abbreviations: APR, abdominoperineal resection; DPC, diagnosis procedure combination; HAR, high anterior resection; Lap, laparoscopic surgery; LAR, low anterior resection; NA, not applicable; NIS, Nationwide inpatient sample; PMS, propensity score matching; Ro, robotic surgery; US, United States.

Our study had several limitations. First, the DPC dataset did not include important information, such as intraoperative and histological outcomes. We could not evaluate the operation time, blood loss, or distance from the anal verge, which have potential impacts on short‐term outcomes. Second, the main diseases in accordance with ICD‐10 codes were defined by the attending surgeons and medical office staff; therefore, the criteria may be open to interpretation. Finally, the learning curves of the surgeons and institutions may not have been considered. Several institutions in Japan performed Ro before 2018, when this surgery was covered by national health insurance. There may be a difference in the short‐term results between these institutions and those that started after 2018. Despite these limitations, this study is the first to report a comparison of the surgical costs of laparoscopic and robotic surgeries using real‐world big data. The accumulation of evidence worldwide is essential to demonstrating the benefits of Ro for rectal cancer.

## 5 CONCLUSION

PSM analysis revealed that Ro was associated with better outcomes than Lap in terms of surgical site infection and respiratory failure rates. The operative medical costs of Ro were significantly higher than those of Lap. However, there was no significant difference in the total medical costs between the two surgeries for rectal cancer.

## AUTHOR CONTRIBUTIONS

**Hiroki Hamamoto:** Conceptualization; project administration; writing – original draft. **Masato Ota:** Formal analysis. **Toru Kuramoto:** Investigation. **Kazuya Kitada:** Investigation. **Kohei Taniguchi:** Investigation. **Mitsuhiro Asakuma:** Investigation. **Yasuhiro Oura:** Investigation. **Yuri Ito:** Supervision.

## FUNDING INFORMATION

The authors received no specific funding for this work.

## CONFLICT OF INTEREST STATEMENT

The authors declare no conflicts of interest for this article.

## ETHICS STATEMENT

Approval of the research protocol by an Institutional Reviewer Board: This study was approved by the Institutional Review Board of Osaka Medical and Pharmaceutical University (approval number: 2020–072‐2).

Informed Consent: N/A.

Registry and the Registration No. of the study/trial: N/A.

Animal Studies: N/A.

## DISCUSSANT

### Professor Yusuke Kinugasa

Thank you for your wonderful presentation and congratulations on receiving the AGSurg Award. Your paper using real world data is very informative for us. And we have some questions for you. First is about the cost. Based on your result, what do you think about the fact that robotic surgery and laparoscopic surgery have the same cost? And furthermore, if additional medical fee point in robotic surgery will be realized, the conclusion will be that robotic surgery will be more expensive. How do you think about it?

### Dr. Hiroki Hamamoto

In this study, I compared the total cost of hospitalization and found no significant difference between the two groups. However, the maintenance cost is higher for the robotic platform than for the laparoscopic platform, so each hospital incurs higher costs for robotic surgery.

Recently, new robotic platforms such as Hugo and HINOTORI have recently emerged. We anticipate that price competition will arise, ultimately leading to a reduction in the cost of robotic surgery. Everything cost related to robot surgery will decrease after price competition, each hospital has to pay less in the future. I think that this situation is similar to when laparoscopic surgery was introduced. Laparoscopic surgery is more expensive than open surgery. Laparoscopic surgery requires a camera system, laparoscopic instruments and surgical ports. Conventional laparoscopic surgery requires 5 ports which were disposal and one port costs at least several thousand yen. Laparoscopic surgery is becoming more popular and less costly, Because of its minimally invasively, proved better outcomes and oncological safety, there is no discussion about cost of laparoscopic surgery nowadays. We believe robotic surgery will be in a similar situation near future.

### Professor Hideki Ueno

First of all, congratulations on winning the award. Dr. Kinugasa put a couple of questions to you about the issue on costs, thus I'll be asking about that on the short‐term outcomes, another part of key conclusions of this paper. Based on the results of the study, you and your colleagues concluded that robotic surgery was more favorable than laparoscope surgery in terms of the short‐term outcomes such as surgical site infection and respiratory complications. But these results may be a little bit counterintuitive for readers. That is because, conventionally, we think that such kind of outcomes are closely relevant to the operative duration which is thought to be longer in the robotic surgery as compared to laparoscopic one. You may have to be addressing the possibility of inaccuracy regarding the data associated with the short‐term outcomes such as respiratory syndrome in the DPC dataset in the Discussion section. In this regard, one possible direction of ours is to combine the DPC database with the National Clinical Database, which would be more accurate in surgical complications, to analyze the correlation between the short‐term outcomes and costs in detail. That may validate your conclusion. But, anyway, your presentation was wonderful, and this paper is very impressive. Congratulations, once again.

### Dr. Hiroki Hamamoto

I completely agree with the suggestion. A major difference between NCD and DPC is hospital scale. As you know, every hospital performing surgery has to participate in the NCD database system, which includes more than 5000 hospitals. In contrast, the DPC insurance system includes around 1700 hospitals. Only one‐third of NCD hospitals use the DPC insurance system. Expert surgeons tend to be more common in DPC‐using hospitals than in NCD‐participating hospitals. Thus, the complication rate tends to be lower in DPC studies than in NCD studies. We want to perform the next study to clarify that point.
